# Identification and validation of neutrophils-related subtypes and prognosis model in triple negative breast cancer

**DOI:** 10.1007/s00432-024-05651-3

**Published:** 2024-03-21

**Authors:** Shanqi Li, Yuzhou Qian, Wanchen Xie, Xinyu Li, Jiaying Wei, Long Wang, Guosheng Ren, Xuedong Yin

**Affiliations:** 1 Department of Breast and Thyroid Surgery, The First Affiliated Hospital of Chongqing Medical University, Chongqing, China; 2 Institute of Life Sciences, Chongqing Medical University, Chongqing, China; 3 Key Laboratory of Molecular Oncology and Epigenetics, The First Affiliated Hospital of Chongqing Medical University, Chongqing, China; 4 Department of Breast Cancer Center, Chongqing University Cancer Hospital, Chongqing, China; 5 Department of Orthopedics, the First Affiliated Hospital of Chongqing Medical University, Chongqing, China

**Keywords:** Neutrophil, Triple-negative breast cancer, Immunotherapy, Tumor microenvironment, Tumor mutation burden

## Abstract

**Background:**

Neutrophils are considered to be crucial players in the initiation and progression of cancer. However, the complex relationship between neutrophils and cancer prognosis remains elusive, mainly due to the significant plasticity and diversity exhibited by these immune cells.

**Methods:**

As part of our thorough investigation, we examined 38 Neutrophils-Related Genes (NRGs) and the associated copy number variations (CNV), somatic mutations, and gene expression patterns in relation to triple negative breast cancer (TNBC). The interactions between these genes, their biological roles, and their possible prognostic significance were then examined. With the NRGs as our basis, we applied Lasso and Cox regression analyses to create a predictive model for overall survival (OS). Furthermore, TNBC tissue and a public database were used to assess changes in MYO1D expression (MYO1D is characterized as a member of the myosin-I family, a group of motor proteins based on actin), its connection to neutrophil infiltration, and the clinical importance of MYO1D in TNBC.

**Results:**

Four neutrophil-related genes were included in the development of a prognostic model based on neutrophils. The model was further shown to be an independent predicted factor for overall survival by multivariate Cox regression analysis. According to this study, neutrophil subtype B as well as gene subtype B, were associated with activated cancer immunity and poor prognosis of TNBC patients. Furthermore, considering that poor OS was linked to increased MYO1D expression, MYO1D was increased in TNBC tissues and associated with neutrophil infiltration. In vitro experiments also confirmed that MYO1D facilitates breast cancer invasion and metastasis.

**Conclusion:**

Based on the degree of gene expression linked to neutrophils, a unique prognostic model was created. MYO1D could be a potential prognostic biomarker in TNBC patients and also a prospective target for therapy.

**Supplementary Information:**

The online version contains supplementary material available at 10.1007/s00432-024-05651-3.

## Introduction

Breast cancer contributes for 31% of the total number of cancer diagnoses in women, resulting in the most common malignancy among them. (Siegel et al. 2023). About 15–25% of instances of newly diagnosed breast cancer are referred to as triple-negative breast cancer (TNBC) (Garrido-Castro et al. 2019). TNBC is inability to produce ER, PR, and HER2 expression makes it resistant to endocrine therapy and anti-HER2 therapy (Won and Spruck 2020). TNBC has a poorer prognosis in contrast to BCs that test positive for HR. In fact, during the first three to five years following diagnosis, about 50% of patients with TNBC have a relapse (Li et al. 2022). Although tumors are heterogeneous and there aren't any other viable long-term therapies outside chemotherapy, TNBC represents the subtype with the poorest prognosis (Bianchini et al. 2022). Promising treatment options have been made available by recent research advances, including as immunotherapy and customized treatment regimens based on genomic sequencing (Yang et al. 2023; Zhu et al. 2021). Treatment treating TNBC using immunotherapy, in particular immune checkpoint inhibitors, has shown some initial success (Schmid et al. 2020). Furthermore, the discovery of BRCA mutations in TNBC patients opens the door to customized medicines that take advantage of certain genetic weaknesses, providing fresh hope for improved therapeutic outcomes (Lyons 2019). Furthermore, genomic profiling-based customized medicine allows for the customization of therapies to the particular features of each patient's tumor. Immunotherapies still do not help most TNBC instances, despite the fact that they do work for some patients. Finding novel biomarkers for the best course of treatment and prognosis prediction has been a constant hot topic in breast cancer research because there are currently no effective therapeutic methods for TNBC.

Neutrophils are important regulators of the innate immune system and chronic inflammation, which is an important component in the development of cancer (Hedrick and Malanchi 2022). Neutrophils play two separate functions in the development and progression of malignancies. Firstly, they can release catalytically active neutrophil elastase (ELANE), which can kill various cancer cell types while sparing non-cancer cells (Xiao et al. 2021; Xiong et al. 2021). Secondly, neutrophils can promote tumor growth and metastasis while simultaneously inhibiting the tumor immune response, leading tumor angiogenesis, and tumor metastasis (Cui et al. 2021; Hajizadeh et al. 2021; Mouchemore et al. 2018; Wculek and Malanchi 2015). Research has repeatedly demonstrated that low clinical outcomes and decreased treatment sensitivity in a variety of cancers are connected with tumor-associated neutrophil infiltration, peripheral blood neutrophil-to-lymphocyte ratios, and neutrophil-based transcriptional signatures (Ocana et al. 2017; Shaul and Fridlender 2019; Szczerba et al. 2019). There is a little study examining the neutrophil-related genes (NRGs) signature in TNBC, despite the proven significance of neutrophils in the advancement of cancer. Furthermore, there hasn't been enough research done on the prognostic significance of neutrophils in particular treatment plans for TNBC. Consequently, to show that particular genes are involved in controlling the growth of TNBC through neutrophil activity, we have carried out studies and bioinformatics research.

The Cancer Genome Atlas (TCGA) database provided us with the NRGs expression data and relevant clinical information of TNBC patients for our research. To differentiate between two different neutrophil subtypes based on their expression levels, we first performed an expression study of NRGs. We next divided the patients into three gene subtypes based on the differentially expressed genes (DEGs) exclusive to these two neutrophil subtypes. We then created a predictive risk model associated to neutrophils to evaluate patient OS (overall survival). Furthermore, we investigated the genetic alterations, biological processes, immunological landscape, and medication susceptibility linked to NRGs in TNBC in detail. Based on the aforementioned investigation, we discovered that MYO1D was significantly infiltrative in neutrophils from triple negative breast cancer and was related to prognosis. Thus, we used transwell, Western Blotting (WB), Real-Time Quantitative Reverse Transcription PCR (qRT-PCR), and Cell Counting Kit-8 (CCK8) assays, respectively, to assess the effect of MYO1D knockdown on the migration, invasion, and proliferation of MDA-MB-231 and BT549 cell lines. We also looked at the relationship between clinical patient survival and MYO1D expression levels.

## Methods

### Data sources

Our data sources were the Gene Expression Omnibus (GEO) database (https://www.ncbi.nlm.nih.gov/geo/) and the TCGA database (https://portal.gdc.cancer.gov/). For further study, RNA expression data for breast cancer were acquired from the TCGA cohort and three GEO cohorts (GSE58812, GSE135565, and GSE45827). This analysis included 1213 samples total from TCGA cohorts (113 normal tissues and 1101 original BC cancers). The First Affiliated Hospital of Chongqing Medical University provided 30 pairs of triple negative breast cancer tissues and the 23 adjacent normal tissue patients (Chongqing, China). Before the procedure, every patient gave their informed consent, and none of them had any other cancers or histories of radiation or chemotherapy. In addition, we obtain from TCGA the mutation, copy number variation (CNV), and pertinent clinicopathological information related to breast cancer. Transcripts per kilobase million (TPM) were created using the fragments per kilobase million (FPKM) values in the RNA sequencing data to facilitate additional research.

### The source of neutrophil-related genes

The neutrophil-related genes TIME-IA (immune activation), TIME-ISM (immune suppressive myeloid), TIME-ISS (immune suppressive stroma), TIME-IE (immune exclusion), and TIME-IR (immune residence) had been identified and divided into five subtypes in a previously published paper (Xue et al. 2022). It was discovered that these subtypes—in particular, TIME-ISM and TIME-ISS—were linked to worse patient outcomes. These two subtypes have also been associated with several biological activities, such as myeloid cell-mediated immune suppression, pro-tumor cytokine release, and angiogenesis stimulation. A total of 38 genes were found to be associated to neutrophils during the investigation and are listed in Table S1 for additional information.

### Functional enrichment, consensus clustering, CNV, and genetic mutation study of NRGs

With the use of the R software packages "maftools" and "RCircos," the somatic mutations of the 38 NRGs were displayed. Using the "ConsensusClusterPlus" packages, consensus clustering was carried out based on patient NRGs expression patterns to create a classification of molecular subtypes for TNBC. To create the heatmaps using the consensus matrix, the maximum number of clusters, K, was set at 9. Next, based on the consensus matrix heatmap and the cumulative distribution function (CDF) curves, the ideal K was ascertained between 2 and 9. Principal component analysis (PCA) was used to confirm that different subtypes of neutrophils have different transcription profiles. The differences between the various subtypes' OS, traits, and NRGs expression levels were compared. Gene set variation analysis (GSVA) was used to examine the signaling pathways associated with the Kyoto Encyclopedia of Genes and Genomes (KEGG) that involved various subtypes. The KEGG curated gene set "c2.cp.kegg.symbols.gmt" was utilized for this purpose (Hänzelmann et al. 2013). Using single-sample gene set enrichment analysis (ssGSEA), the number of immune cells invading the TNBC tumor microenvironment (TME) was measured.

### Determination of prognostic and differentially expressed NRGs in neutrophil subtypes

To find NRGs that were expressed differently in TNBC samples compared to normal samples, the "limma" package was utilized. Using the "survival" and "survminer" packages, Kaplan–Meier curves were used to determine the relationships between the patients' OS and NRGs expression. The "limma" software was utilized to calculate DEGs between various neutrophil subtypes, requiring a log2-fold change greater than 1.5 and an adjusted p-value less than 0.05. Utilizing KEGG pathway analysis with the "clusterProfifiler" package and Gene Ontology (GO) annotation, functional enrichment of these subtype-related DEGs was carried out.

### The neutrophil-related prognostic risk model's construction

First, using univariate COX regression with *P* < 0.05, the prognostic-related NRGs were found. Next, using unsupervised clustering analysis, we separated the samples into distinct gene clusters based on the expression of prognostic-related NRGs. The "caret" R software was then used to randomly split all the data into training and testing sets in a 1:1 ratio. Additionally, the neutrophil-related prognostic risk model was then constructed in the training set. Both the external cohort and the testing group provided validation for the risk model. Using the R package "glmnet," LASSO regression was used to avoid over-fitting and track each variable's trajectory. Finally, utilizing multivariate COX regression analysis, the independent prognostic-related genes were ultimately eliminated. Using the "rms" R package, we constructed a nomogram using risk-score. The calibration curve was used to evaluate the model's correction, and the time-dependent area under the ROC curve (AUC) was used to evaluate the model's discrimination. The patients were divided into low-risk and high-risk groups based on the training set's median risk score. Using the R package "dplyr," a Sankey diagram was created to display the cluster distribution with various risk groups and survival outcomes.

### Integrated analysis of tumor immune, CSC index, tumor mutation, and drug susceptibility

For the tumor immune analysis, we employed the CIBERSORT method. Initially, we looked into the relationship between risk score and genes linked to prognosis using 21 immune cells that infiltrate tumors. Then, using the "Estimate" R package, we computed TME scores—which included stromal, immune, and estimate scores—for both high- and low-risk groups. We also looked into the connection between risk score and stemness scores. Using the "GENE" module, which is based on the TIMER database, the degree of immune cell infiltration in three genes was evaluated. (https://cistrome.shinyapps.io/timer/) (Li et al. 2016, 2017). Using the "maftools" R package, we transformed the somatic mutation file that was taken from the TCGA database into the mutation annotation format (MAF) and checked the mutation status of samples belonging to the high- and low-risk categories. Additionally, we determined the tumor mutation burden (TMB) score for each of the two risk groups and looked into the relationship between the TMB and risk scores. Lastly, we computed the semi-inhibitory concentration (IC50) values of widely used chemotherapeutic medications for TNBC using the "pRophetic" R package, and we compared the variations in chemotherapeutic drug efficacy between high- and low-risk groups.

### Cell culture and transfection

MCF-7 and SK-BR-3 were preserved in DMEM High Glucose supplemented with 1% penicillin and streptomycin and 10% fetal bovine serum (FBS). RPMI 1640 medium containing 10% FBS, 1% penicillin, and 1% streptomycin was used to cultivate T-47D, BT-549, MDA-MB-231, and MDA-MB-468. In F12 media supplemented with 10% FBS and 1% penicillin and streptomycin, MCF10A was cultivated. Gibco provided the medium, FBS, and Penicillin–Streptomycin (Gibco-BRL, Karlsruhe, Germany). Every cell was kept in a 37 °C environment with 5% CO_2_ MYO1D small interfering RNA (siRNA) knockdown with the help of a pool of siRNA duplexes (ONTARGETplus SMARTpool, Dharmacon), the MYO1D gene was temporarily silenced. In accordance with the manufacturer's instructions, the siRNA was transfected into the designated cells using Lipofectamine 2000 (Invitrogen; Thermo Fisher Scientific, Inc., USA). To confirm the effectiveness of the interference, qRT-PCR and a Western blotting experiment were then performed.

### RNA isolation and real-time qRT-PCR

Following the manufacturer's instructions, total RNA was isolated from cells using Trizol reagent (Takara, Beijing, China), and reverse transcription into complementary DNA was performed using the PrimeScriptTM RT reagent kit with genomic DNA eraser (Takara, Beijing, China). SYBR Green qPCR Master Mix (MedChemExpress, Monmouth Junction, NJ, USA) was used for the qRT-PCR study. The internal reference gene was GAPDH. A variety of primers were utilized in the qRT-PCR analysis. The 2^− ∆∆CT^ technique was used to determine the mRNA levels. Triples of the samples were examined.

### Immunohistochemical (IHC) and tissue microarray (TMA) analyses

Thirty TNBC samples from various instances made up TMAs. The First Affiliated Hospital of Chongqing Medical University's Research Ethics Committee gave its approval for the gathering of samples. The sections underwent heat-induced antigen retrieval, deparaffinization, rehydration, blocking, and washing. An Envision/horseradish peroxidase system (Dako-Cytomation, Glostrup, Denmark) was used to identify immunoreactive signals. Hematoxylin was used as a counterstain for each segment, and each was examined under a light microscope. The KM plotter database (http://kmplot.com) assesses the MYO1D genes' survival in TNBC. (Győrffy 2021).

### Transwell assays

Transwell chambers (1492309x, Aabselect, Beijing, China) equipped with an 8 μm pore filter and matrigel (Abwbio, Shanghai, China) were used for the cell migration and invasion assay. To perform the migration test, 3 × 104 cells were seeded with 200 uL of serum-free 1640 medium in the upper chamber of a 24-well plate, and 500 μL of 1640 media containing 20% FBS was added to the lower chamber. The cells were then grown with this mixture for six hours. Following the proper incubation period, migrating cells were fixed for 30 min with 4% paraformaldehyde and stained for 30 min with 0.1% crystal violet. Subsequently, using cotton swabs to remove any cells that remained in the top chamber, wash with 1 × PBS. To conduct the invasion assay, 70 μL of diluted Matrigel was dripped into the upper chamber and allowed to solidify at 37 °C. The remaining procedures were the same as described before. This was done around 4 h before the experiment started. Five randomly selected fields were counted under an inverted microscope (100 ×) (OLYMPUS CKX53, Japan) to determine the invasive cell count.

### Cell viability assay

After 48 h of transfection, MDA-MB-231 and bt-549 cells in the logarithmic growth phase were plated in a 96-well plate (5 × 10^3^ cells/well) and let to develop for 24, 48, and 72 h. Subsequently, the cells were processed according to the manufacturer's instructions using the CCK8 kit after being incubated for one hour with 10 μL of CCK8 solution (Beyotime Biotechnology, Shanghai, China). The Varioskan Flash (Thermo Scientific, USA) was utilized to record the absorbance of the reaction mixture at 450 nm.

### Western blot

48 h after transfection, cell lysates were prepared, and the protein content was measured using an improved BCA protein Assay kit (Beyotime, Shanghai, China). After heating the proteins to denature them, 10% SDS-PAGE was used to separate them. Transferred on PVDF membranes (0.45 µm, Merck Millipore, Germany) were the isolated proteins. After blocking, primary antibody incubation, washing, and visualization with an upgraded chemiluminescence detection system (Tanon 5200, China), the membrane was used. GAPDH served as an internal safeguard. Three separate Western blot analyses were carried out.

### Statistical analysis

A statistically significant value was defined as a two-sided probability value of *p* < 0.05. RStudio (version 4.2.2) was used for data processing and visualization.

## Results

### Genetic expression and mutation of NRGs

**Fig. 1:**
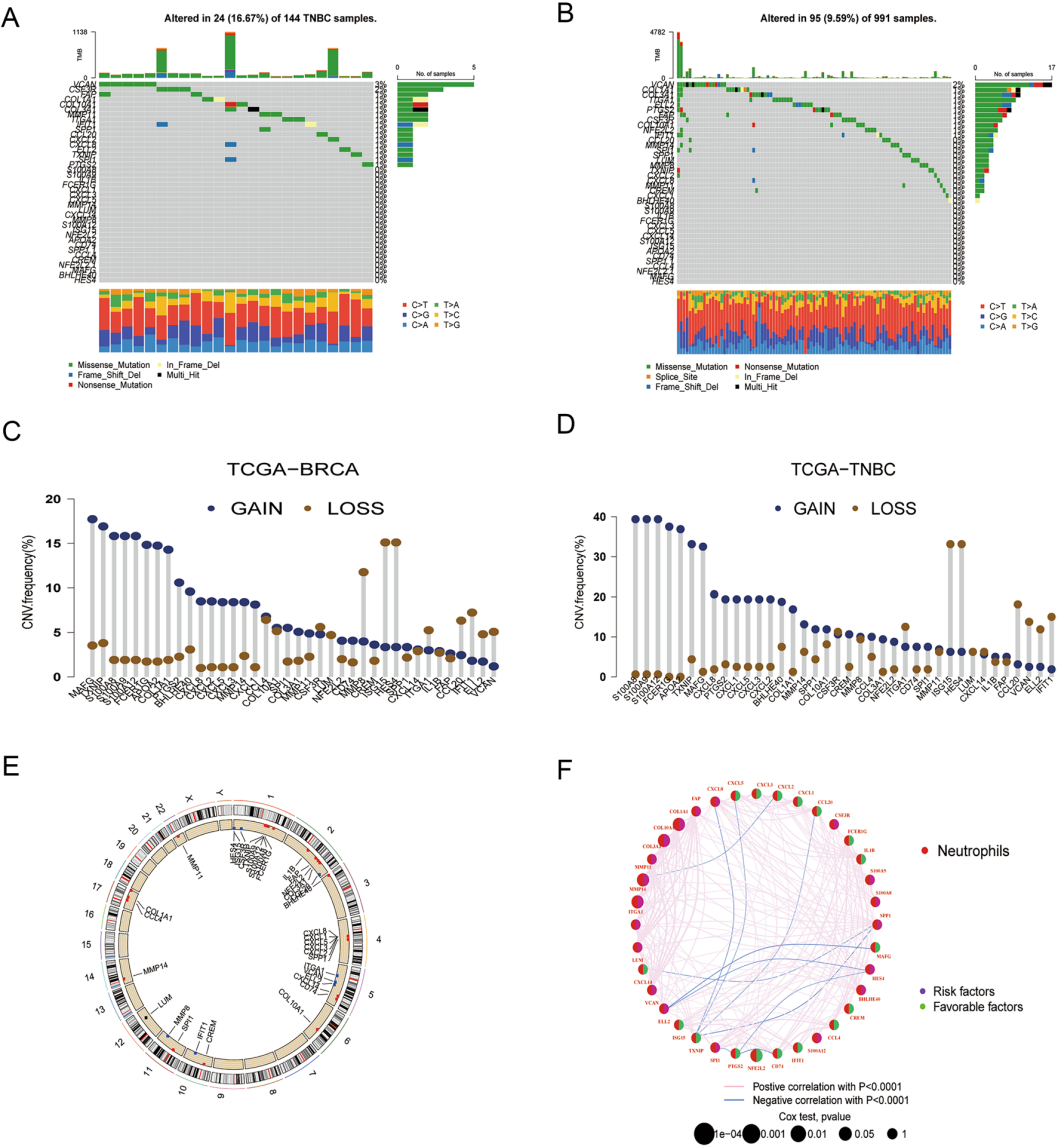
Genetic variations of NRGs in BC and TNBC. **A**, **B** Mutation frequencies of 38 NRGs in BC samples and TNBC samples. **C**, **D** Figures represent the CNV alteration frequency of NRGs in BC samples and TNBC samples. **E** Locations of CNV alterations in NRGs on chromosomes. **F** The interaction and prognostic significance of NRGs across all 304 TNBC samples. *NRGs* neutrophil-related genes, *BC* breast carcinoma, *TNBC* triple-negative breast cancer, *CNV* copy number variation

We analyzed somatic mutations in 38 NRGs in our investigation, and the results showed that patients with TNBC had a significantly greater mutation frequency (16.67%) than did all the cohorts of breast cancer (BC) combined (9.59%) (Fig. 1A, B). Remarkably, missense mutations were the most common form of mutation found in VCAN, making it the most frequently mutated NRGs.

In addition, we looked into copy number variants (CNVs) in all 38 NRGs in patients with TNBC (Fig. 1C) as well as in individuals in the larger BC patient population (Fig. 1D). S100A8 showed the highest frequency of copy number increase among TNBC patients, closely followed by S100A9 and S100A12. On the other hand, we found that among TNBC patients, copy number losses were more common in ISG15 and HES4. Every BC patient exhibited distinct patterns of CNV, with MAFG showing the highest occurrence of increasing CNV and ISG15 and HES4 showing the most frequent decreases in CNV (Fig. 1E) shows the precise sites of copy number variations in NRGs on chromosomes in TNBC patients.

To look into differentially expressed NRGs, RNA sequencing data from 113 TNBC samples and 113 normal tissues from TCGA-BRCA were employed. Of the 38 NRGs, 36 showed different expression levels between cancer and normal tissues (Fig. S1). We used COX univariate regression analysis to examine the association between NRGs and breast cancer OS and to create a survival curve for each gene based on a *p* < 0.05 cutoff to investigate the impact of NRGs on tumorigenesis (Fig. S2). Next, we demonstrated the interactions, mutual regulation, and prognostic significance of these NRGs in patients with breast cancer using a neutrophil network (Fig. 1F).

### Analyzing TME characteristics and identifying neutrophil subgroups in TNBC

**Fig. 2:**
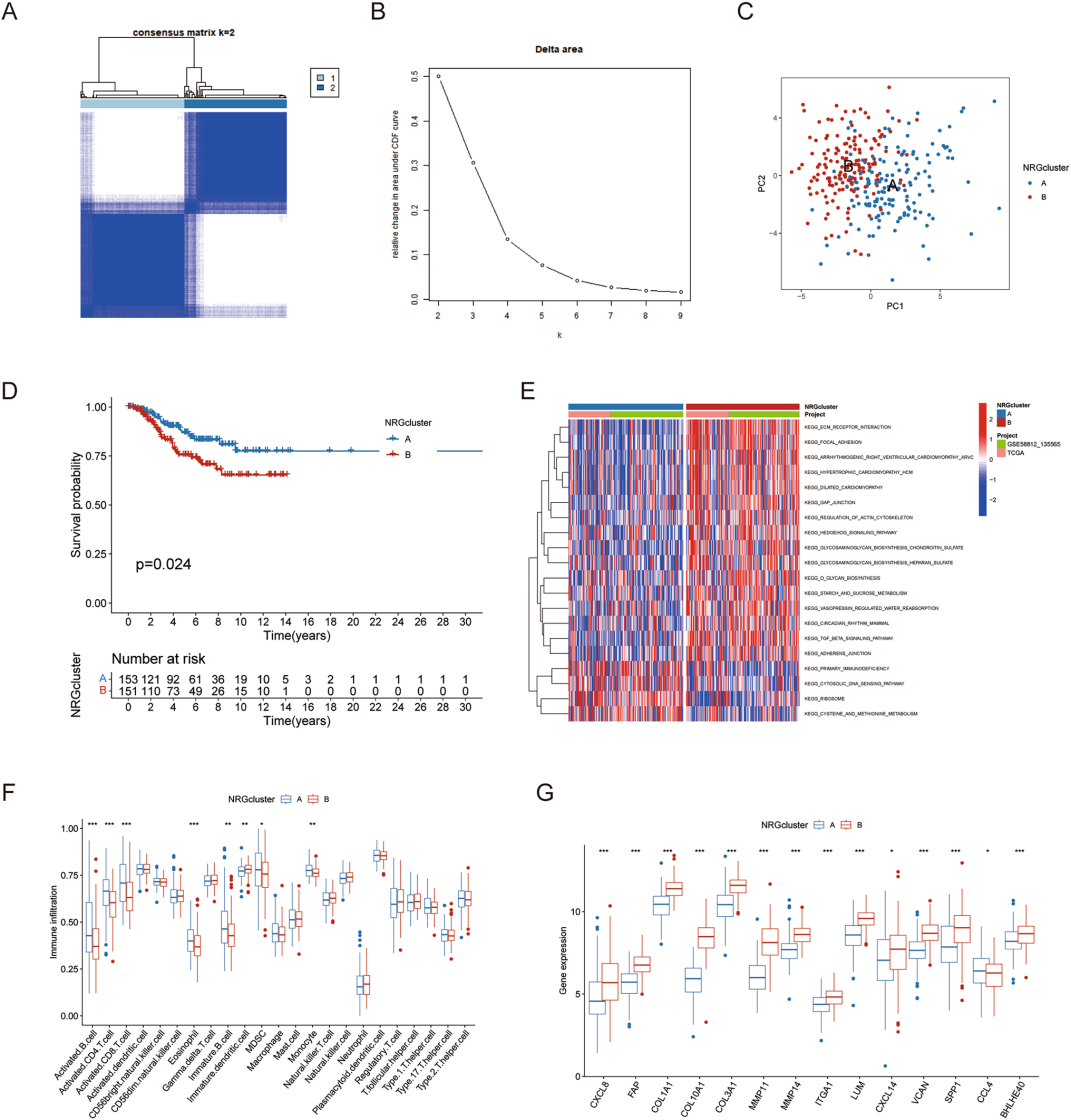
Clustering analyses and TME features of neutrophil subtypes in TNBC. **A**, **B** Consensus matrix heatmap defining two neutrophil-related subtypes (k = 2). **C** PCA analysis demonstrates a distinctive difference between the two clusters. **D** Kaplan–Meier OS curves for the two clusters. **E** GSVA pathway enrichment analysis of two types of NRGs. **F** eight immune cell types showed distinctly higher infiltration levels in the microenvironment of subtype. **G** Comparisons of NRG expression in two PRG subtypes. *p < 0.05, **p < 0.01, ***p < 0.001

For consensus clustering analysis, the NRGs expression profile of 304 TNBC samples from the development cohort was retrieved. The subtype clustering includes 36 NRGs that were found in the TCGA-BRCA, GSE58812, and GSE58812 datasets (Table S2). The TNBC samples were divided into groups A (*n* = 153) and B (*n* = 151) based on the heatmap of the consensus matrix and the CDF curves, which determined that *k* = 2 was the ideal group number (Fig. 2A, B). The unique characteristics of the neutrophil transcriptomes of the two groups were confirmed by PCA (Fig. 2C). Additionally, patients of subtype A had a significantly higher OS compared to those of subtype B (*p* = 0.024) according to Kaplan–Meier curves (Fig. 2D).

Subtype B was found to have a significantly higher enrichment in extracellular matrix, including ECM-receptor interaction, focal adhesion, TGF beta signaling, Gap junction, regulation of actin cytoskeleton, Hedgehog signaling, adherens junction, glycosaminoglycan biosynthesis chondroitin sulfate, glycosaminoglycan biosynthesis heparan sulfate, and primary immunodeficiency, according to GSVA analysis of the canonical pathway of 304 TNBC samples. Seven of the twenty-three immune cell types that were examined had significantly larger infiltration levels in subtype A's TME than in subtype B's, according to ssGSEA (Fig. 2F). Differential NRG expression was found between gene subtypes, which is in line with the neutrophil-based categorization (Fig. 2G). Ultimately, an analysis of the NRG expression in each cluster revealed a significant difference in the expression of NRGs. These findings indicated that patients with TNBC of subtype B had a higher propensity for tumor spread.

### DEG-based gene subtype formation and DEG identification among neutrophil subtypes

**Fig. 3:**
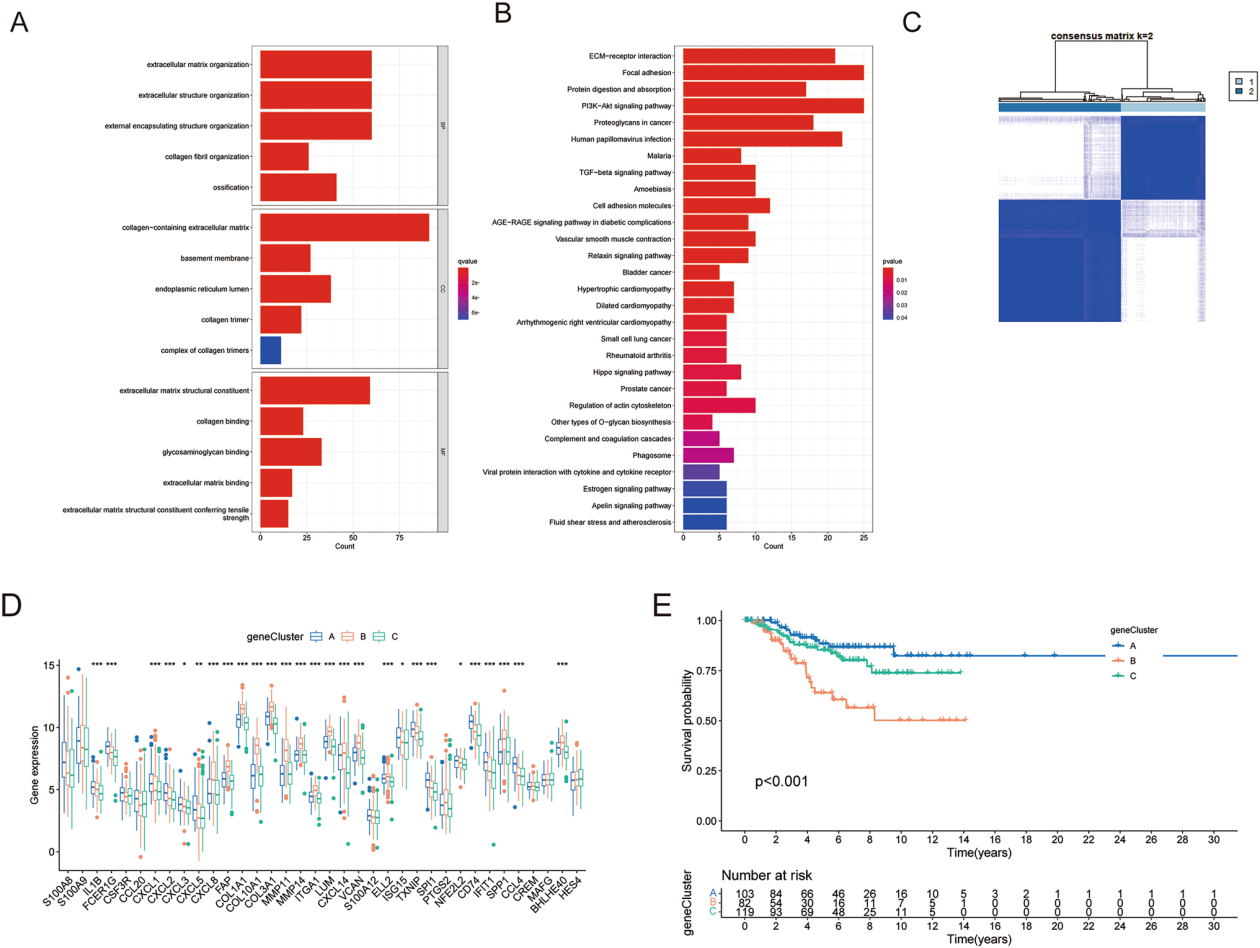
Development of gene subtypes based on differentially expressed genes between NRG subtypes. **A**,**B** GO and KEGG enrichment of DEGs identified between two NRG subtypes. **C** Consensus matrix heatmap defined three gene subtypes. **D** NRGs were differentially expressed between three gene subtypes. **E** TNBC patients defined as gene subtype C showed significantly worse OS. *p < 0.05, **p < 0.01, ***p < 0.001

After eliminating 340 DEGs associated with neutrophil subtypes, we carried out a functional enrichment analysis (Table S3, Fig. 3A, B). These subtype-related DEGs were linked to biological processes such collagen fibril organization, extracellular matrix organization, extracellular structure organization, and external encapsulating structure organization, according to GO analysis. These NRGs were mostly linked to the ECM-receptor interaction, focal adhesion, protein digestion and absorption, PI3K-Akt signaling pathway, proteoglycans in cancer, and other processes, according to KEGG pathway analysis.

To assess the predictive value of each of the 340 DEGs in TNBC, we ran univariate COX regression on them. Based on *p* < 0.05, we identified 71 genes for additional investigation (Table S4). The cohort was then split into three gene subtypes using a consensus clustering approach based on these prognosis-related genes (Fig. 3C). At last, an analysis of the NRG expression in each gene cluster revealed a significant difference in the expression of NRGs (Fig. 3D). According to the Kaplan–Meier survival analysis, gene cluster B had the worst prognosis (Fig. 3E) and the OS among the three gene subtypes was significant (*p* < 0.001).

### Development of a risk score using prognostic DEGs associated to neutrophils.

**Fig. 4:**
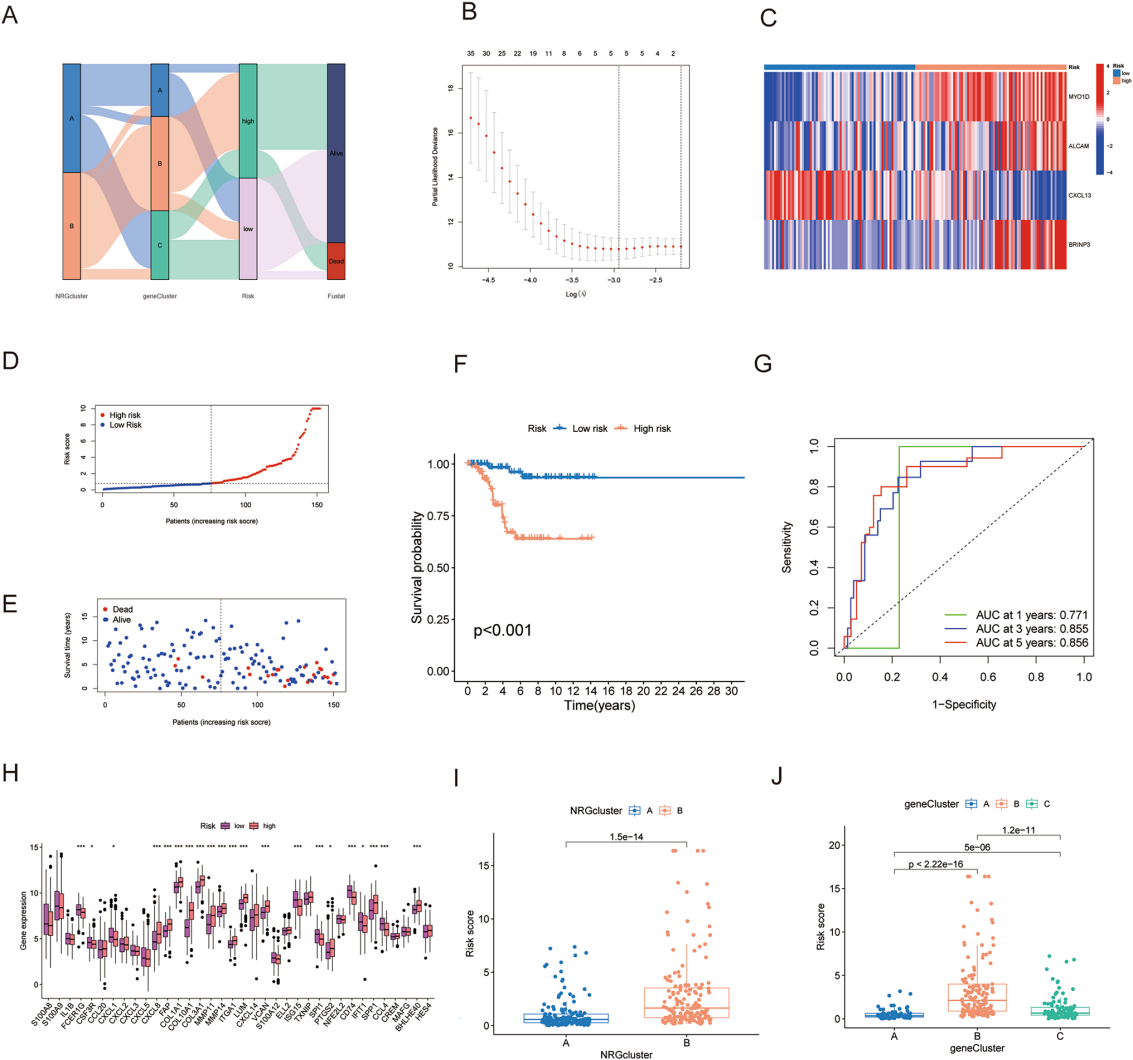
Construction of a risk score based on neutrophil-related prognostic DEGs. **A** Sankey diagram of samples distribution between different subtypes, risk groups, and vital status. **B** Lasso regression analysis on the prognosis-related genes. **C** Expression heatmap of the 4 hub DEGs between two risk groups. **D** Risk score distribution in patients from training set. **E** Vital status plot showed higher death rate in patients from high-risk group in training set. **F** TNBC patients from high-risk group had markedly worse OS than low-risk patients. **G** ROC curves to predict1-, 3-, and 5-year OS according to the risk-score in the training cohort. **H** The differential analysis of NRGs expression (**I**) and the NRGs cluster (**J**). *p < 0.05, **p < 0.01, ***p < 0.001

In the risk model exploration, 354 instances from the development cohort (TCGA-BRCA, GSE58812, and GSE135565) were included. The patients' distribution across several subtypes, risk groups, and vital status is depicted in Fig. 4A's alluvial diagram. A training set (*n* = 152) and an internal validation set (*n* = 152) were randomly assigned to the patients. Using LASSO regression and cross-validation, we identified 5 potential genes based on the patient survival results and the expression of 71 prognostic DEGs associated with neutrophils (Fig. 4B; Fig. S3). In the end, we established the risk score with the coefficients and expression levels of the three DEGs (COL5A3, ALCAM, and BRINP3) using multivariate Cox regression analysis. To create a risk model (Table S5), we finally filtered out four genes: MYO1D, ALCAM, BINP3, and CXCL13. These genes turned out to be significant predictors of OS in patients with TNBC. Figure 4C shows the expression heatmap of the four DEGs between the two risk groups.

The threshold value to differentiate between high- and low-risk patients was determined to be a median score of 0.8008 in the training set (Fig. 4D). Patients in the high-risk group died at a higher rate than those in the low-risk group, according to the vital status plot (Fig. 4E). Significantly worse OS in high-risk individuals was corroborated by the Kaplan–Meier curves (Fig. 4F). The ROC curve analysis revealed that the 1-, 3-, and 5-year OS predictions had respective areas under the curves (AUCs) of 0.771, 0.855, and 0.856 (Fig. 4G). The testing cohort's AUC values were 0.513, 0.637, and 0.668, while the overall cohort's 1-, 3-, and 5-year values were 0.579, 0.740, and 0.762 (Fig. S4). Additionally, among all patients in the development cohort, a difference in the expression of 36 NRGs was found between the high- and low-risk groups (Fig. 4H). Patients from both neutrophil subtype B and gene subtype B, which represented activated cancer immunity and worse OS, had a significantly higher risk score than those from neutrophil subtype A and gene subtype A, respectively (*p* < 0.001 in both tests) (Fig. 4I, J), out of the 304 individuals in the entire development cohort. This suggests that the lower risk score may be linked to an upregulated immune defense in the TNBC microenvironment.

### Assessment of the tumor immune milieu according to risk classification

**Fig. 5:**
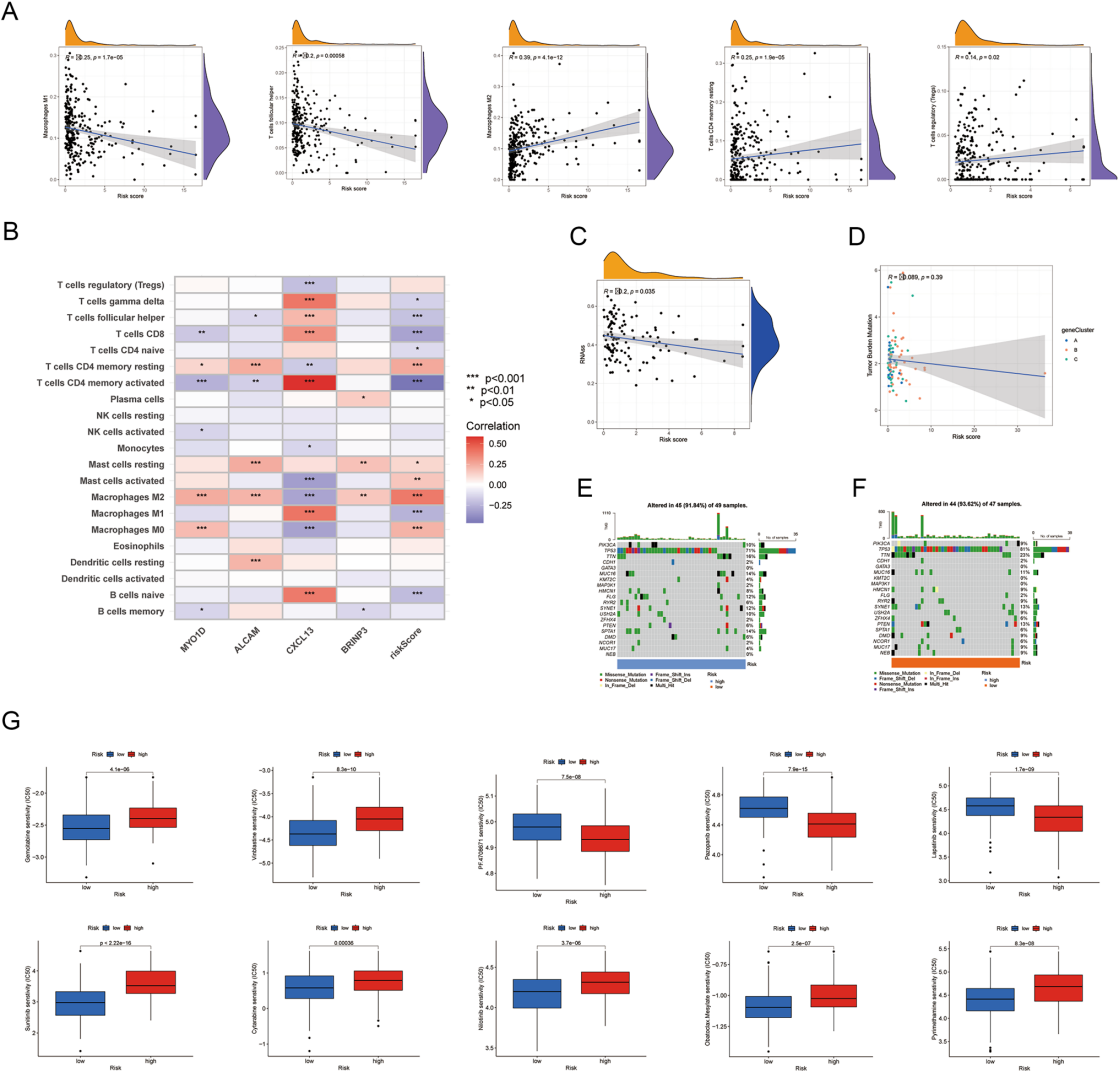
Characteristics of the TME, analyses of somatic mutation, CSC index and drug susceptibility based on risk stratification. **A** Association between risk score and immune cell infiltration. **B** Association between the abundance of immune cells and four genes in the risk score model. **C** Comparison of TMB between risk groups. **D** Correlation between risk score and CSC index. **E**, **F** The waterfall plot of somatic mutation features in the high-risk group (**E**) and low-risk group (**F**). **G** Drug susceptibility analyses (IC50) to assess the response differences to commonly used chemotherapy reagents between patients classified as high-risk and low-risk. *TME* tumor microenvironment, *CSC* cancer stem cell, *IC50* half-maximal inhibitory concentration. *p < 0.05, **p < 0.01, ***p < 0.001

We used the CIBERSORT algorithm to examine the relationship between immune cell abundance and risk score to investigate tumor immunity and the microenvironment in TNBC from various risk groups. The risk score was inversely correlated with four types of antitumor immune cell types (M1 macrophages, T cells CD4 memory activated, T cells gamma delta, and T cells follicular helper) and positively correlated with the fraction of six activated or pro-tumorigenic cell types (NK cells activated, T cells regulatory (Tregs), Mast cells resting, Mast cells activated, T cells CD4 memory activated, and Macrophages M2). At least one of the four DEGs in the scoring model showed a statistically significant link with the 21 immune cell types that were examined (Fig. 5B). The discovery that low-risk patients were more likely to belong to immune-activated subtypes was supported by the results of low-risk score samples with high abundance of antitumor immune cells.

The TME (Tumor Microenvironment) score is comprised of three parts, which we calculated using the "ESTIMATE" package: an immune score that indicates the degree of immune cell infiltration, a stromal score that indicates the presence of stromal cells in the tumor tissue, and an ESTIMATE score that is a composite score that is derived from the stromal and immune scores and provides an estimate of tumor purity. According to our study, TNBC patients in the low-risk category scored higher on each of the three components (Fig. S5). The higher scores indicate a reduced level of tumor purity, which is attributed to a greater influx of immune and stromal cells into the tumor microenvironment. Furthermore, we discovered from the internet database that there was a positive correlation between MYO1D and neutrophil infiltration in TNBC (Fig. S6).

### Mutation and drug sensitivity analysis

First, we analyzed somatic mutations in two different risk categories: high and low. The gene with the highest frequency of mutations in both risk groups was TP53. On the other hand, we noticed differences in gene mutations between these groups. Particularly, it was discovered that the low-risk group had more mutations in genes like PIK3CA and MMUC16 than the high-risk group. On the other hand, as Fig. 5E, F show, TTN showed a greater mutation frequency in the high-risk group compared to the low-risk group. Notably, there were no discernible variations seen in any of the three groups when we looked at the tumor mutational burden (TMB), as shown in Fig. 5D. Moreover, we also looked at any possible relationships between the risk scores and the CSC index values. The CSC index and risk score show a negative connection in Fig. 5C, suggesting that TNBC cells with lower risk scores have more evident stem cell characteristics. To wrap up our investigation, we assessed the TNBC patients' vulnerability to widely utilized treatment drugs in both the high- and low-risk groups.

To determine the best course of action for TNBC patients, we assessed the chemotherapeutic medicines' susceptibility in high- and low-risk situations. While low-risk patients in low-risk groups had lower IC50 values for pyrimethamine and obatoclax.mesylate, high-risk patients showed greater sensitivity to pazopanib, lapatinib, and bicalutamide (Fig. 5G). According to susceptibility testing, high-risk patients may benefit more from medications like vinblastine and gemcitabine.

### MYO1D was up-regulated in triple negative breast cancer

**Fig. 6:**
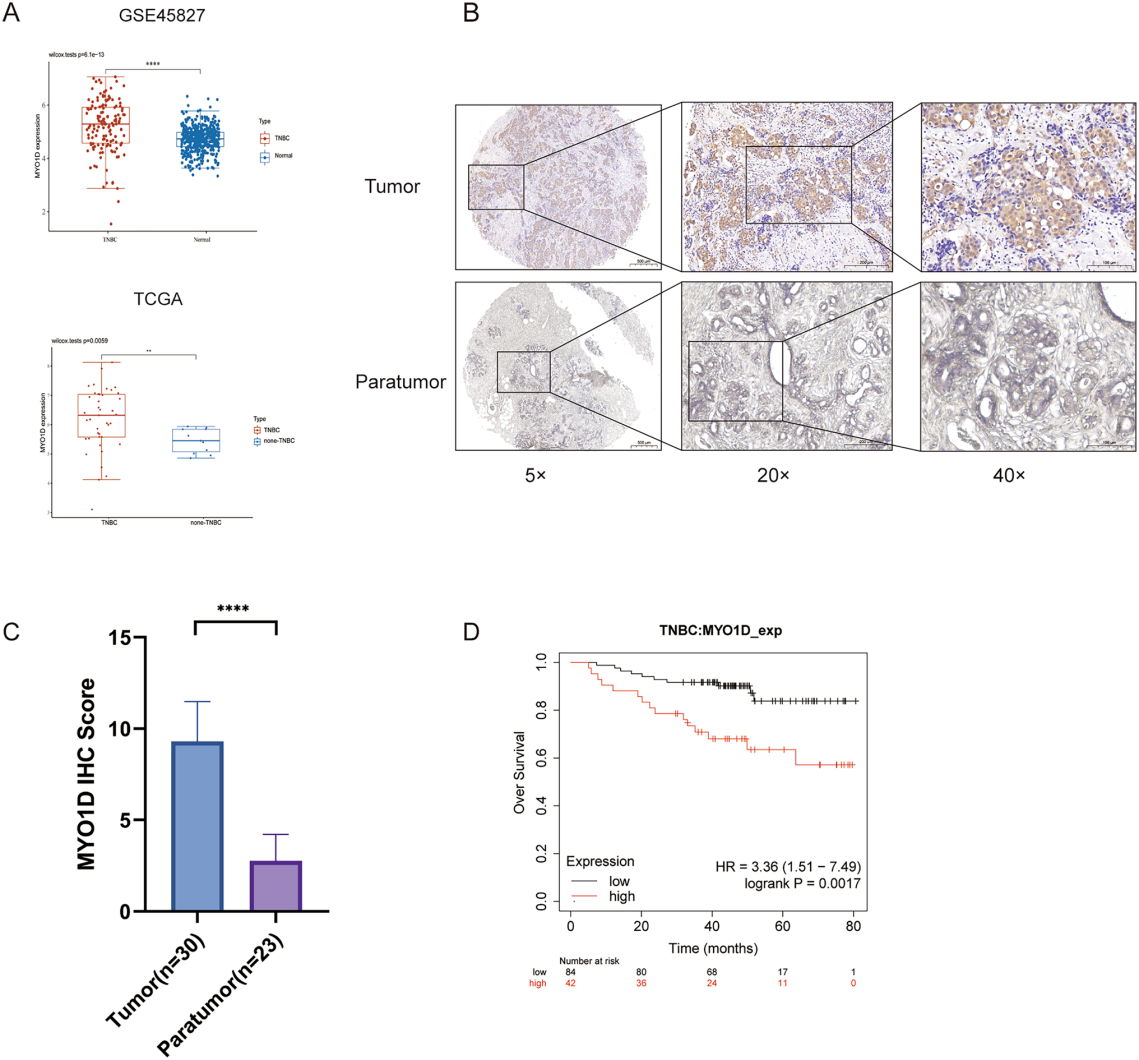
The different expression of MYO1D in TNBC. **A** The scatter plots of MYO1D mRNA expression levels in patients with triple-negative breast cancer in GSE45827 database and TCGA databases. **B** Representative images of IHC staining results of MYO1D in TNBC tissues and adjacent normal tissues. **C** The IHC score analysis of MYO1D expression levels in paired breast cancer compared to matched normal tissues in our included patients' tissues. **D** Kaplan–Meier analysis of overall survival of TNBC patients according to the expression of MYO1D. P-values in **A** were calculated using Wilcox test. P-value in **C** was calculated using paired two-tailed Student's t tests. *TNBC* triple negative breast cancer, *IHC* immunohistochemistry, *GEO* gene expression omnibus; *p < 0.05; **p < 0.01; ***p < 0.001; ****p < 0.0001

The TNBC RNA expression data, which included TCGA-TNBC and GSE45827, were obtained from the TCGA and GEO databases. Compared to patients with non-triple-negative breast cancer, patients with triple-negative breast cancer had greater levels of MYO1D expression (Fig. 6A). Additionally, the expression of MYO1D was examined by immunohistochemistry staining in 30 pairs of breast cancer and 23 paratumor tissues. The findings demonstrated that, in comparison to matched nearby normal tissues, MYO1D expression was higher in cancerous tissues (Fig. 6B, C). Moreover, poor overall survival (OS) was linked to increased MYO1D expression, according to TCGA data (Fig. 6D).

### Effects of MYO1D on the malignant biological behavior of TNBC cells

**Fig. 7:**
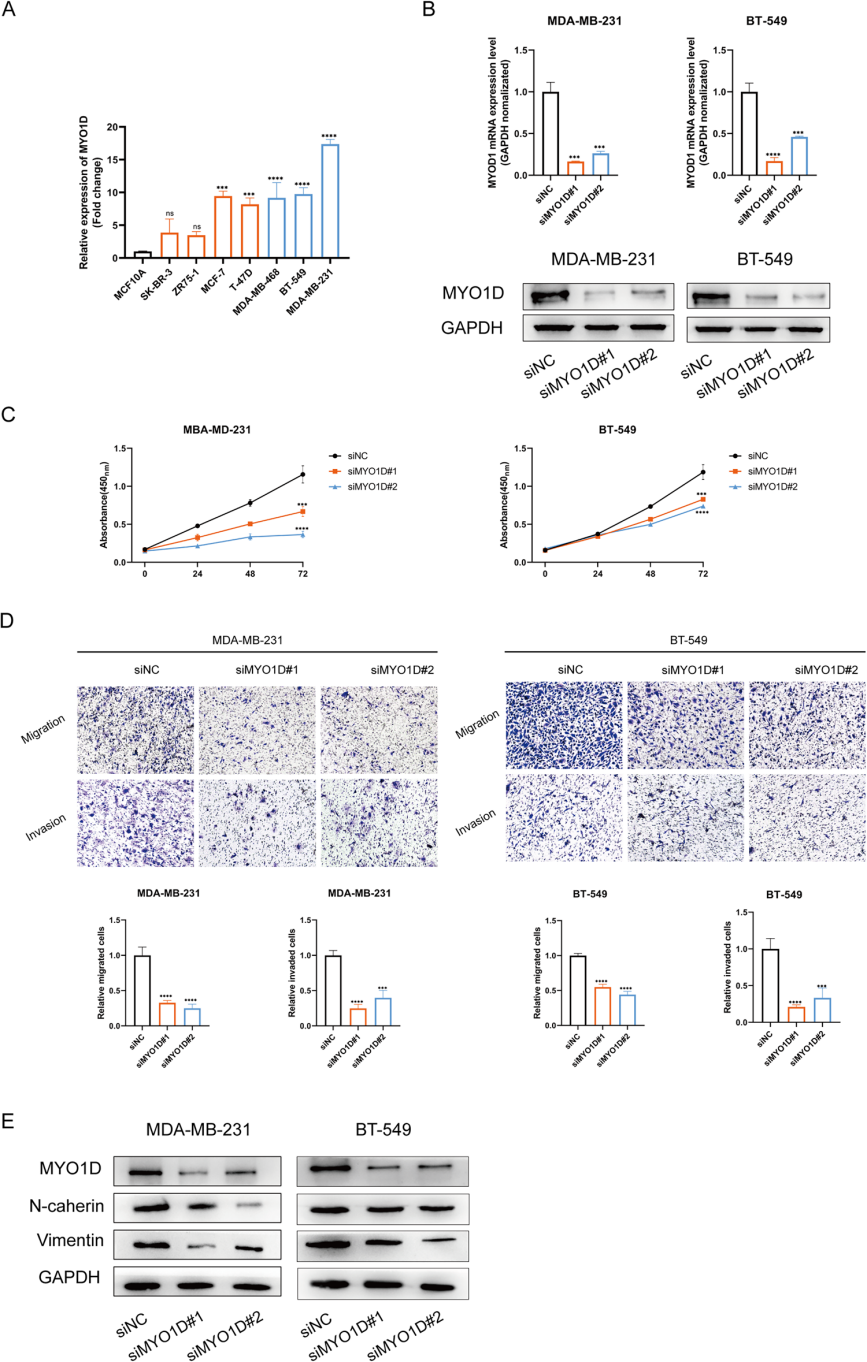
MYO1D promotes the invasion and metastasis of breast cancer cells in vitro. **A**, **B** Expression of MYO1D mRNA (**A**) and protein (**B**) was examined in 8 kinds of cells by qRT-PCR and Western blotting. **C** The changes in proliferation capacity of MDA-MB-231 and BT-549 cells after knocking down MYOD1 were verified by CCK-8 experiments. **D** In Transwell experiments, the impact of MYO1D knockdown on the invasive and migratory capabilities of MDA-MB-231 and BT-549 cells was assessed. **E** The expression of MYOD1 and EMT correlated factors was verified by WB experiments. *EMT* epithelial cell-to-mesenchymal transition, *qRT-PCR* quantitative polymerase chain reaction, *CCK-8* cell counting kit-8 *p < 0.05; **p < 0.01; ***p < 0.001; ****p < 0.0001

We examined MYO1D mRNA levels in several cell lines to clarify the function of MYO1D in breast cancer metastasis. Figure 7A shows that MCF-7, T-47D, MDA-MB-468, BT-549, and MDA-MB-231 had greater levels of MYO1D expression. Simultaneous copies of MDA-MB-231 and BT-549 were transfected. Western blot and qRT-PCR were used to determine the MYO1D knockdown efficiency (Fig. 7B). The MDA-MB-231 and BT-549 breast cancer cell lines' vitality was assessed using the CCK-8 assay after MYO1D knockdown to examine the function of MYO1D. After MYO1D knockdown, both cell lines' viability dropped (Fig. 7C). Transwell experiments demonstrated that MYO1D knockdown also reduced the capacity for cell migration and invasion in MDA-MB-231 and BT-549 cells, which is consistent with these findings (Fig. 7D). After knocking down MYO1D, the protein levels of N-cad and vimentin were reduced in MDA-MB-231 and BT-549 cells, which inhibited EMT (epithelial cell-to-mesenchymal transition) in breast cancer cells in comparison to the control group (Fig. 7E).

## Discussion

This research constitutes a ground-breaking attempt to comprehensively look into and compile data on NRGs in TNBC. We, therefore, performed a thorough investigation of neutrophil-related genes in TNBC, taking into account gene expression levels, somatic mutations, CNVs, immunological infiltration, TME, CSCs, treatment sensitivity, and other relevant variables. We classified TNBC samples into two different neutrophil subtypes—referred to as Subtype A and Subtype B—based on our study of a collection of forty NRGs. Subtype A showed better overall survival results. We used gene set variation analysis (GSVA) and gene set enrichment analysis (GSEA) to acquire a deeper understanding of the biological processes and immune cell infiltration connected to these two categories. According to our research, Subtype B was very abundant in pathways including TGF-β signaling, focal adhesion, protein digestion and absorption, PI3K-Akt signaling, and ECM-receptor interaction. Additionally, compared to Subtype A, Subtype B showed a greater level of immune cell infiltration. We were able to distinguish three separate gene subtypes by finding genes that were expressed differently between these neutrophil subtypes. Based on these discoveries, we created a risk score model to forecast TNBC patients' overall survival (OS). Our study found that TNBC patients classified as having low-risk and high-risk NRG scores differed significantly in terms of prognosis, mutation profiles, TME features, CSC indices, and medication sensitivity profiles. All things considered, our findings highlight the potential value of NRGs as useful markers for prognostic evaluation and immunotherapy response prediction in TNBC.

Historically, breast cancer has been described as immune-cold; however, new research indicates that TNBC may have an immunocompetent subtype that may be targetable with immunotherapeutic treatments (Thorsson et al. 2018). Even with improvements in immunological protocol and targeted therapy, the prognosis for TNBC is still not good. Thus, to maximize illness care, useful prognostic and therapeutic markers are required. Furthermore, the contribution of TME to the genesis and development of breast cancer has been ignored by conventional prediction techniques, which have mostly focused on the intrinsic characteristics of cancer (So et al. 2022). TANs are essential to the TME, and in recent years, there has been a growing awareness of their significance in the advancement of cancer (Coussens and Werb 2002). Through a number of processes, such as the stimulation of angiogenesis, the induction of immunosuppression, and the promotion of genomic instability, TANs most likely aid in the creation and propagation of tumors (Que et al. 2022; Xiong et al. 2021). Neutrophils have the potential to influence the development of breast cancer either directly or indirectly. This is mainly due to their propensity to increase inflammatory responses, promote angiogenesis, and mediate immunosuppressive effects. Furthermore, neutrophils have been linked to the prognosis and responsiveness to treatment for breast cancer (Hajizadeh et al. 2021; Zheng et al. 2023). In individuals with breast cancer, a high infiltration of TANs inside the tumor parenchyma has been linked to a poor prognosis and a worse progression-free survival rate (Sheng et al. 2023). Neutrophils may play a role in TNBC cell migration, according to in vitro investigations (SenGupta et al. 2021).

We used the TCGA and GEO datasets to perform an analysis to shed further light on the connection between neutrophils and the prognosis of TNBC. Additionally, we discovered three gene subtypes and created a risk_score model for OS prediction in TNBC based on the DEGs between the two neutrophil subtypes. There has never been any research done on the relationship between neutrophil-associated genes and the prognosis of TNBC. As a result, this study looked at TNBC prognosis and drug therapy prediction independently using genes linked to neutrophils. Moreover, we confirmed in vitro the association between the malignant biological behavior of TNBC cells and the variable expression in the risk model. The outcomes of the bioinformatics study aligned with the in vitro experiment results.

Of the chosen gene signatures, MYO1D has been found to have increased expression levels in breast cancer and has the capacity to upregulate the expression of the Epidermal Growth Factor Receptor (EGFR), which improves the motility and viability of colorectal and breast cancer cells (Ko et al. 2019). As a motor protein based on actin, MYO1D is a member of the myosin-I family and performs a variety of tasks in the cell, including helping to transport and provide mechanical support for membrane-bound organelles as well as participating in the processes of exocytosis and endocytosis. For instance, MYO1D is responsible for capturing and retaining proteins within specific organelles or subcellular regions (McConnell and Tyska 2010; Sanz-Moreno and Marshall 2010). In addition, MYO1D has drawn interest as a potential new therapeutic target, especially when combined with EGFR Tyrosine Kinase Inhibitors (TKIs) to block it. This combined method shows promise as a successful treatment option for patients with glioblastoma and lung cancer with various EGFR mutations, including those who have developed resistance to osimertinib in Non-Small Cell Lung Cancer (NSCLC) (Ko et al. 2021). Furthermore, prior studies have demonstrated that MYO1D interacts with SPAG6 and regulates the expression of the EGFR family, signaling pathways, and the advancement of acute murine leukemia (AML). This implies that MYO1D might be a viable treatment target for AML (Mu et al. 2022). In this investigation, we found that MYO1D and neutrophil infiltration in TNBC were positively correlated based on our review of online databases. We discovered an increase of MYO1D in TNBC tissues through in vitro investigations. Moreover, our results verified that MYO1D stimulates breast cancer invasion and metastasis.

Apart from assessing immune cell infiltration, tumor mutation burden (TMB) evaluation has become a viable biomarker for prognosticating and therapy response in a variety of cancers (Zhang et al. 2020; Zhu et al. 2020). VCAN mutations had the highest frequency in both risk groups according to our data, which is consistent with findings from previous research. According to studies, VCAN mutations may increase TMB, which would improve the objective reactions to immune checkpoint blockade treatments (Wang et al. 2022). Treatment effectiveness and prognosis are closely correlated with TMB, with higher TMB typically translating into better survival rates (Liu et al. 2022; Marabelle et al. 2020). Our data revealed that the low-risk group had superior survival rates and greater TMB levels. It is crucial to recognize, nonetheless, that the very small sample size used in this investigation may have reduced the statistical significance of these results. To sum up, TMB and particular gene alterations like VCAN have the potential to be important markers for prognosis and therapy responses in TNBC. However, it is crucial to understand that the sample size employed in this analysis may have limited the statistical robustness of our findings. It is necessary to conduct larger-scale research projects to validate and improve these findings.

Compared to other breast cancer subtypes, the TNBC subtype has a higher incidence of CSC characteristics, which could explain why it is more invasive and susceptible to metastasis (Bai et al. 2018; Zhang et al. 2023). Prior research has indicated that TNBC's treatment resistance may be attributed to CSCs (Nasr et al. 2018; Palomeras et al. 2018). We found that there was a negative relationship between the CSC index and the neutrophils risk score in our investigation. Furthermore, our investigation of drug sensitivity showed that the low-risk group had lower half-maximal inhibitory concentrations (IC50) of conventional chemotherapeutic drugs, such as vinblastine and gemcitabine, than the high-risk group did. These results highlight the potential importance of targeted CSC therapy in reducing metastasis and improving TNBC patient survival (He et al. 2021).

Our study is the first multiomic analysis to explore neutrophil-associated risk genes in triple-negative breast cancer and confirmed that high expression of MYO1D leads to a poor prognosis in TNBC. Nevertheless, it is necessary to acknowledge the many limitations of our investigation. Our study demonstrated a significant association between the tumor microenvironment and genes related to neutrophils. However, the underlying process is yet unknown, thus more research is required to fully comprehend this association. Consequently, future analyses should use a more current and comprehensive set of neutrophil-related genes to maintain the validity of the research.

## Conclusions

The purpose of this study was to look into how NRGs might affect many elements of TNBC development and therapy, such as drug sensitivity, prognostic values, immune infiltration, expression profile, mutation, and CNV. We were able to create a predictive model that emphasizes the function of NRGs in both immunotherapy and targeted therapy by performing a thorough investigation of NRGs. In this study, the association between the B subtype of neutrophils and the B subtype of genes was observed in relation to heightened cancer immunity activation and an unfavorable prognosis in patients with TNBC. Additionally, the heightened expression of MYO1D was correlated with diminished OS. Furthermore, MYO1D was shown to be overexpressed in TNBC tissues. In vitro, MYO1D has been shown to facilitate the invasion and metastasis of breast cancer. Consequently, MYO1D emerges as a potential prognostic biomarker in TNBC patients and holds promise as a prospective therapeutic target.

## Supplementary Information

Below is the link to the electronic supplementary material.

Supplementary file 1 (DOCX 9206 kb)

Supplementary file 2 (XLSX 9 kb)

Supplementary file 3 (XLSX 9 kb)

Supplementary file 4 (XLSX 12 kb)

Supplementary file 5 (XLSX 9 kb)

Supplementary file 6 (XLSX 9 kb)

## Data Availability

Any reasonable request can be made to the corresponding author to have access to any datasets used and/or analyzed during this investigation.
