# Correction: Identification and validation of neutrophils-related subtypes and prognosis model in triple negative breast cancer

**DOI:** 10.1007/s00432-024-06048-y

**Published:** 2024-12-16

**Authors:** Shanqi Li, Yuzhou Qian, Wanchen Xie, Xinyu Li, Jiaying Wei, Long Wang, Guosheng Ren, Xuedong Yin

**Affiliations:** 1 Department of Breast and Thyroid Surgery, The First Affiliated Hospital of Chongqing Medical University, Chongqing, China; 2 Institute of Life Sciences, Chongqing Medical University, Chongqing, China; 3 Key Laboratory of Molecular Oncology and Epigenetics, The First Affiliated Hospital of Chongqing Medical University, Chongqing, China; 4 Department of Orthopedics, The First Affiliated Hospital of Chongqing Medical University, Chongqing, China; 5 Department of Breast Cancer Center, Chongqing University Cancer Hospital, Chongqing, China

**Correction to: Journal of Cancer Research and Clinical Oncology (2024) 150:149**

10.1007/s00432-024-05651-3

In the original version of this article, Figur 7E contained duplicate images in the internal control (GAPDH) for the MDA-MB-231 and BT-549 cells. The image has now been corrected to use the correct data.


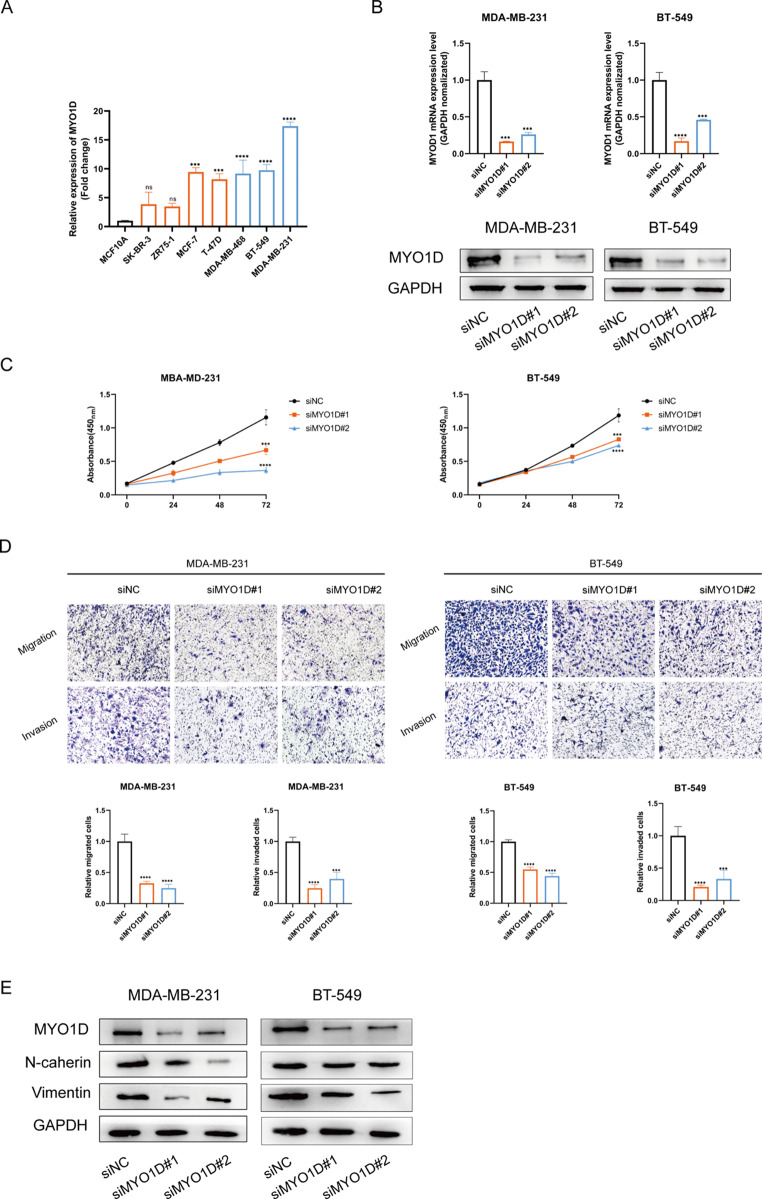


The original article has been updated.

