# Supplementary material for: Identification and validation of neutrophils-related subtypes and prognosis model in triple negative breast cancer
Source: J Cancer Res Clin Oncol. 2024 Mar 21;150(3):149. doi: 10.1007/s00432-024-05651-3 (PMC10957690; doi:10.1007/s00432-024-05651-3)
Supplement: Supplementary file 1 — (DOCX 9206 kb) [file 432_2024_5651_MOESM1_ESM.docx]

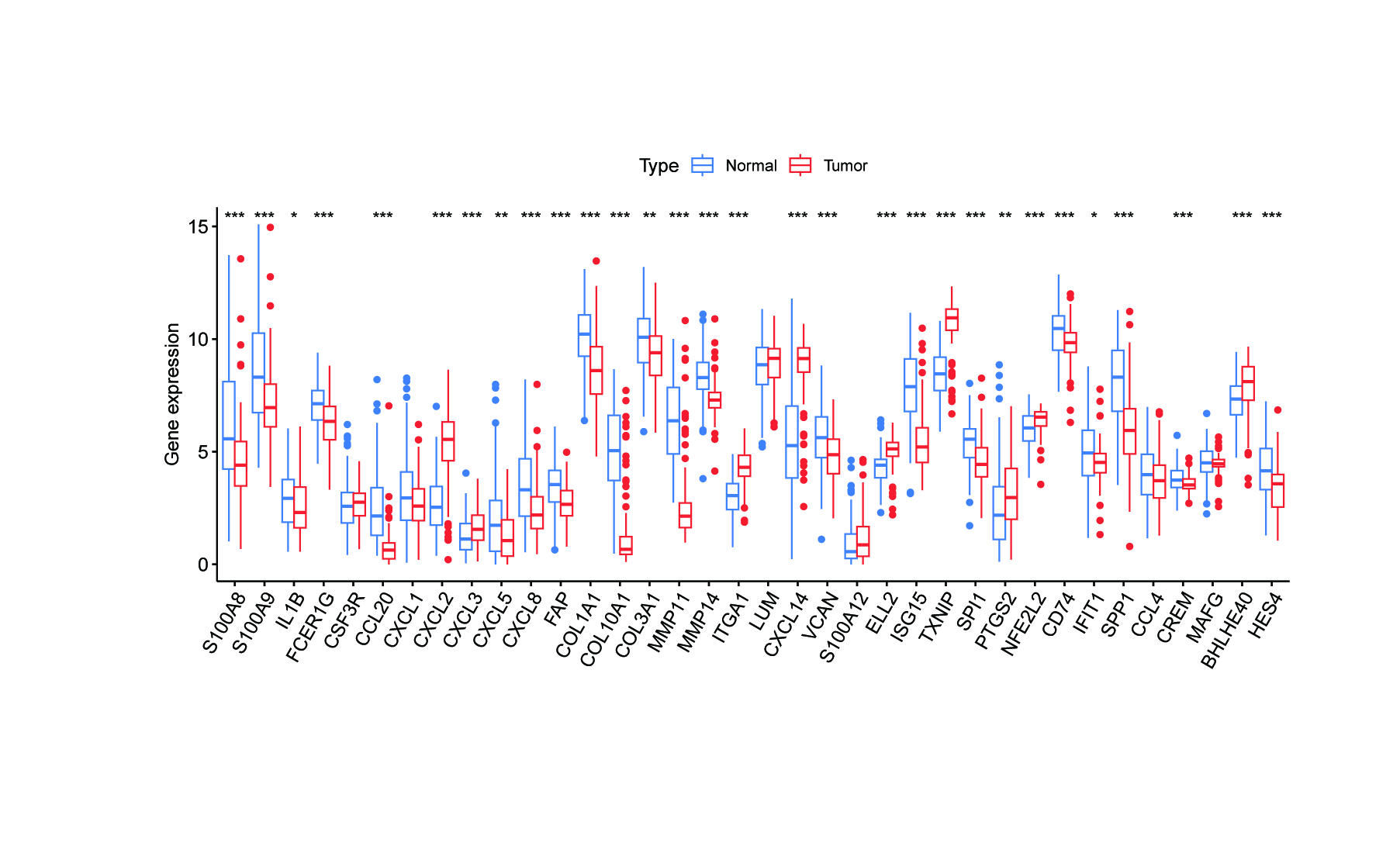


Supplementary Figure S1

Differential expression of neutrophil-associated genes in triple-negative breast cancer.


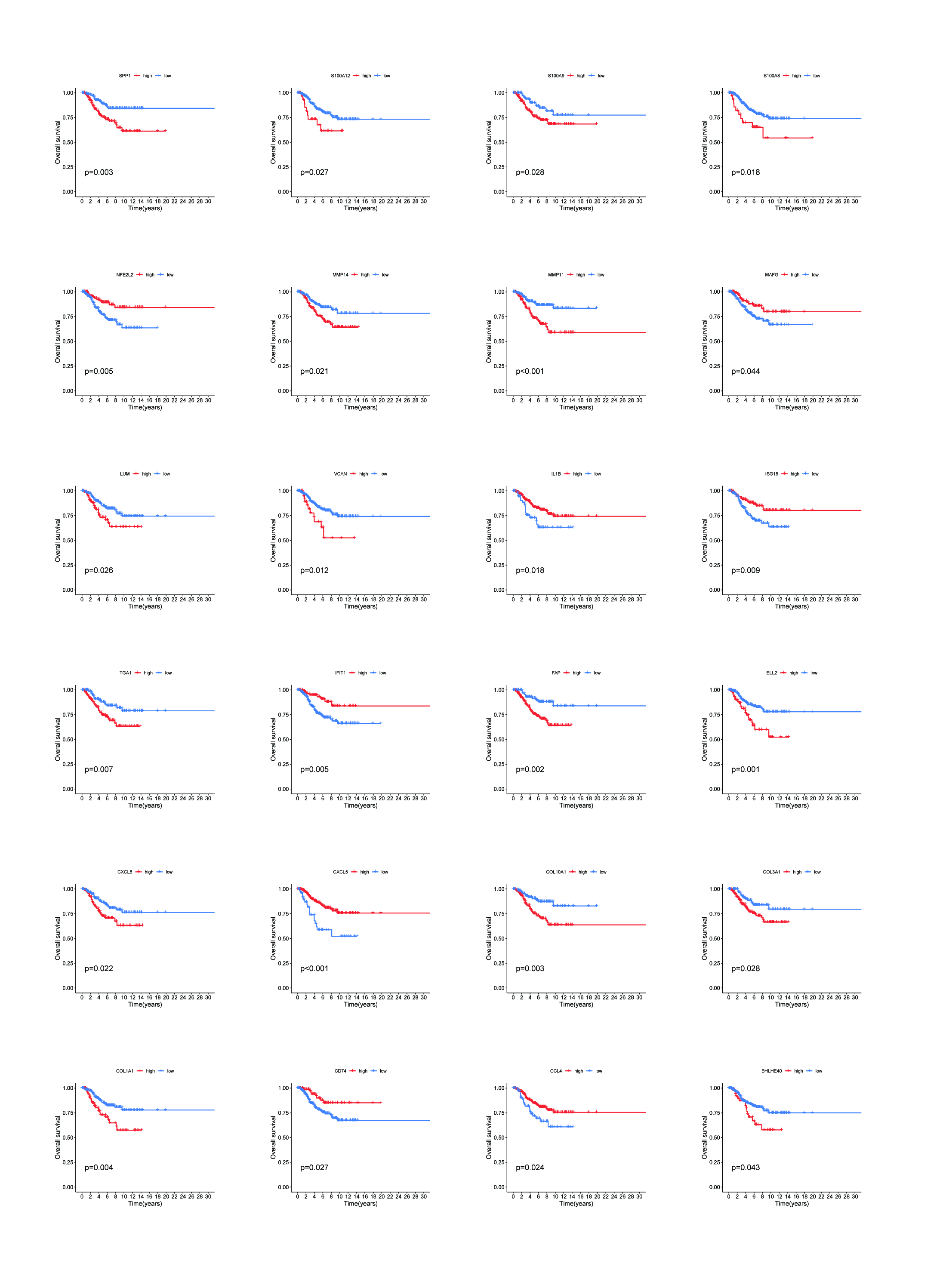


Supplementary Figure S3
twenty-four OS-related NRGs in TNBC.


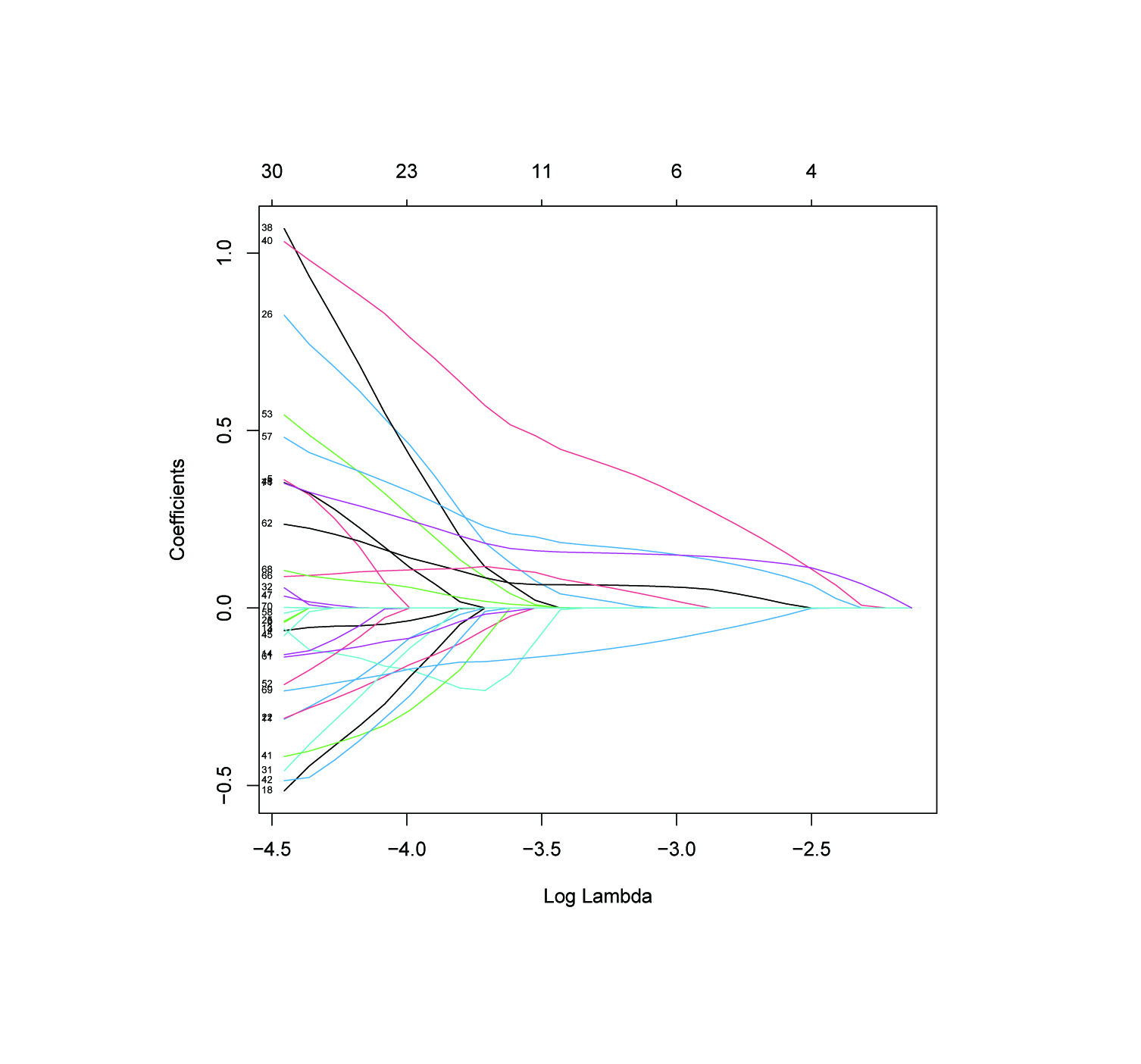


Supplementary Figure S3

Lasso regression analysis on the prognosis-related genes.


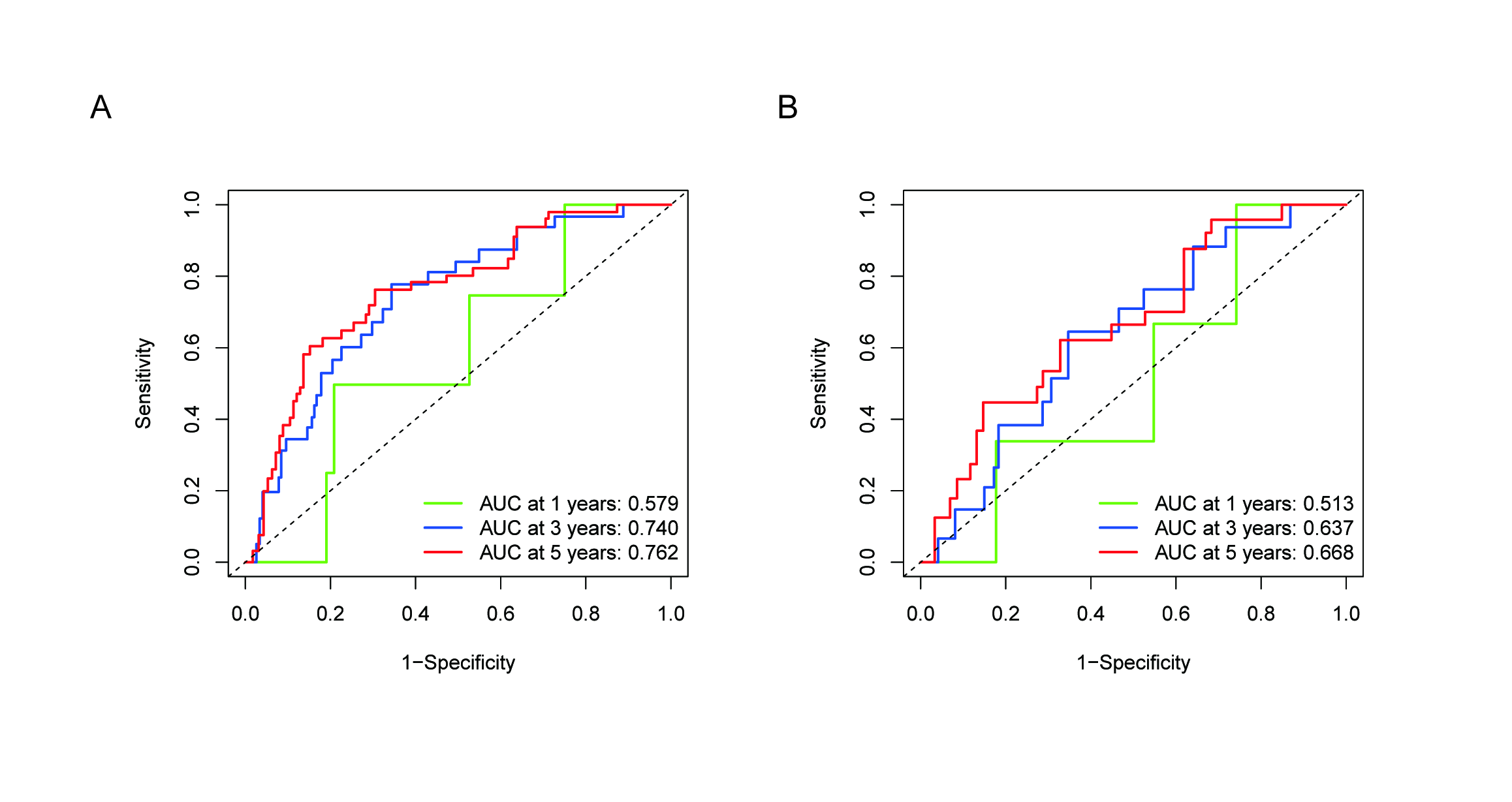


Supplementary Figure S4

ROC curves predicted the sensitivity and specificity of the 4-gene-based risk score in predicting 1-, 3- and 5- year OS for patients in internal validation set.


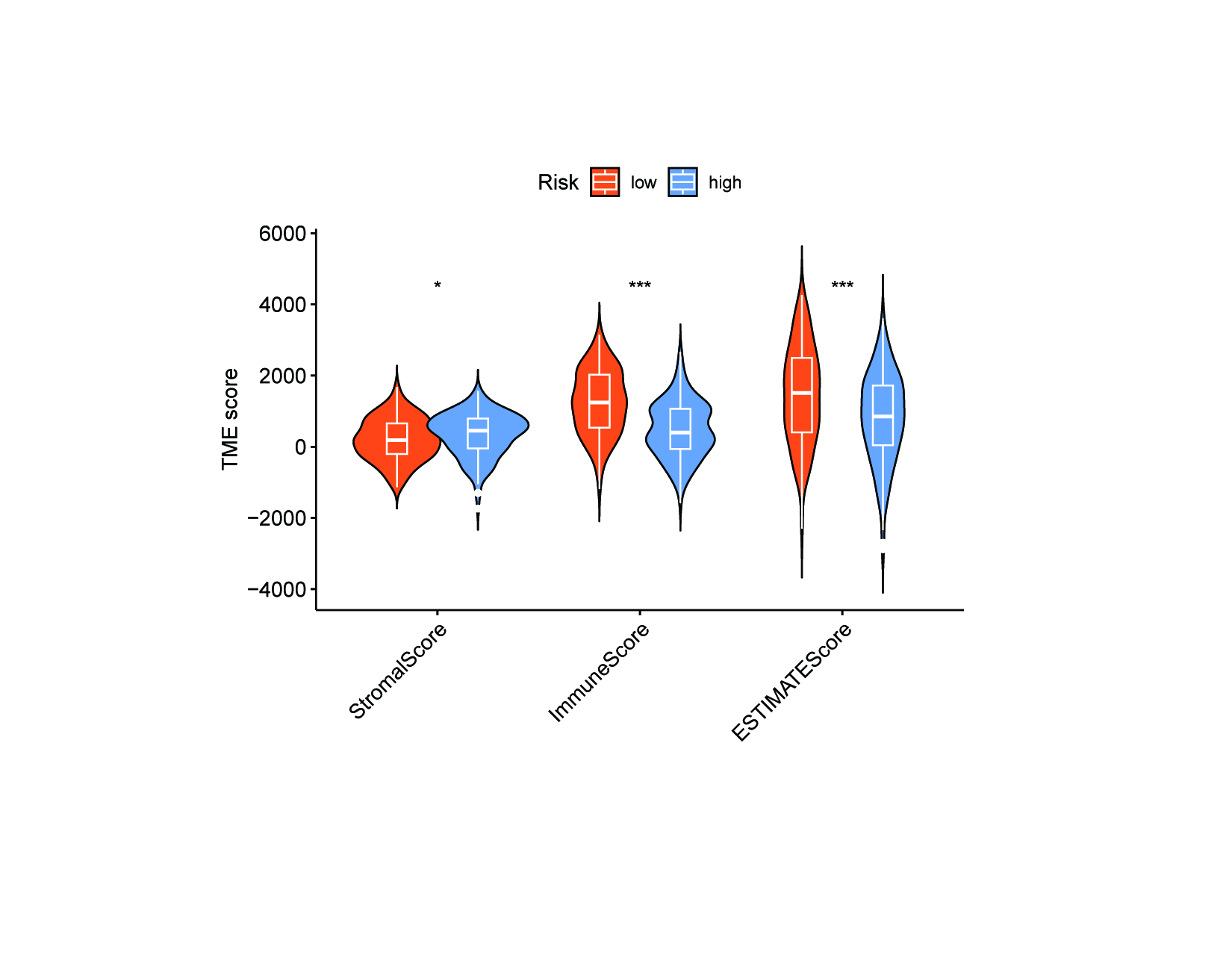


Supplementary Figure S5

TNBC samples from low-risk patients showed higher stromal, immune, and ESTIMATE scores, indicating lower tumor purity with more stromal cells and immune cells in TME.


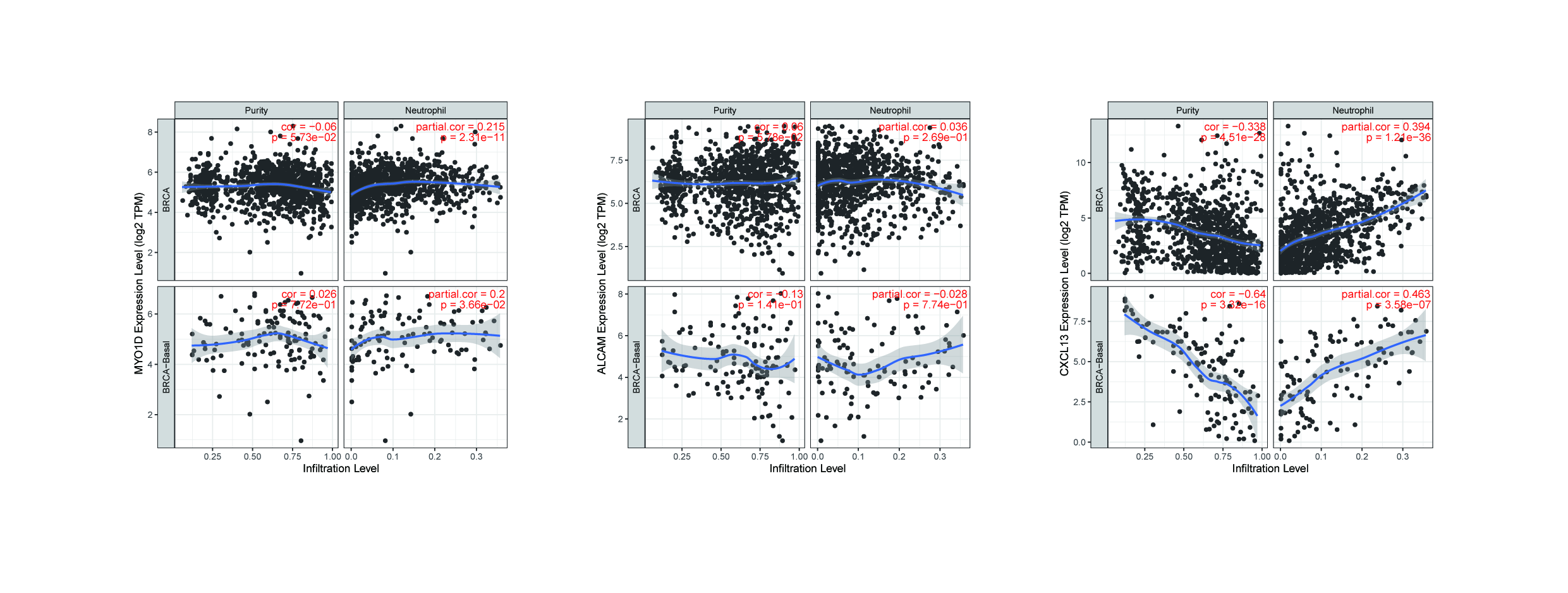


Supplementary Figure S6

Scatterplots show correlation of three genes with neutrophil invasion in breast cancer and TNBC.
